# Phasor Fluorescence Lifetime Microscopy of Free and Protein-Bound NADH Reveals Neural Stem Cell Differentiation Potential

**DOI:** 10.1371/journal.pone.0048014

**Published:** 2012-11-05

**Authors:** Chiara Stringari, Jamison L. Nourse, Lisa A. Flanagan, Enrico Gratton

**Affiliations:** 1 Laboratory of Fluorescence Dynamics, Biomedical Engineering Department, University of California Irvine, Irvine, California, United States of America; 2 Department of Neurology, Sue & Bill Gross Stem Cell Research Center, University of California Irvine, Irvine, California, United States of America; Indian Institute of Toxicology Reserach, India

## Abstract

In the stem cell field there is a lack of non invasive and fast methods to identify stem cell’s metabolic state, differentiation state and cell-lineage commitment. Here we describe a label-free method that uses NADH as an intrinsic biomarker and the Phasor approach to Fluorescence Lifetime microscopy to measure the metabolic fingerprint of cells. We show that different metabolic states are related to different cell differentiation stages and to stem cell bias to neuronal and glial fate, prior the expression of lineage markers. Our data demonstrate that the NADH FLIM signature distinguishes non-invasively neurons from undifferentiated neural progenitor and stem cells (NPSCs) at two different developmental stages (E12 and E16). NPSCs follow a metabolic trajectory from a glycolytic phenotype to an oxidative phosphorylation phenotype through different stages of differentiation. NSPCs are characterized by high free/bound NADH ratio, while differentiated neurons are characterized by low free/bound NADH ratio. We demonstrate that the metabolic signature of NPSCs correlates with their differentiation potential, showing that neuronal progenitors and glial progenitors have a different free/bound NADH ratio. Reducing conditions in NPSCs correlates with their neurogenic potential, while oxidative conditions correlate with glial potential. For the first time we show that FLIM NADH metabolic fingerprint provides a novel, and quantitative measure of stem cell potential and a label-free and non-invasive means to identify neuron- or glial- biased progenitors.

## Introduction

Current label-based methods used to describe cell phenotype have to date proven inadequate for accurately predicting the differentiation potential of many stem cell populations. A technique to detect the potential of NPSCs to generate either neurons or glia would improve the use of these cells in therapies.

The ability to direct differentiation of NPSCs towards neurons and to identify neuron-restricted progenitor cells may provide new therapeutic avenues for stroke, spinal cord injury and age-related cognitive conditions, such as Alzheimer’s and Parkinson’s diseases, which cause loss of neurons. The mammalian brain contains a population of neural stem cells (NSCs), which can self renew and differentiate to give raise to neurons, astrocytes and oligodendrocytes. They are relatively quiescent in adults [1], entering the cell cycle to produce more rapidly dividing progenitors that undergo limited rounds of proliferation and are more committed to specific neural lineages [2]. Astrocytes perform many different functions, including providing structural and nutrient support for neurons, secreting signaling molecules, and uptake and metabolism of neurotransmitters.

Traditional cell sorting is performed by flow cytometry or fluorescence-activated cell sorting (FACS) that provide separation of cellular populations based on fluorescent labeling of cell surface markers [3]. For example different surface markers have been identified for NSPCs (CD133, SSEA-1 [CD15], A2B5), and differentiated neurons (CD24, NCAM, CD56) [4], [5]. Although cell sorting efficiency has been optimized in the last years [6], [7], [8], [9], [10] cell viability after sorting is still not very high and the capability of sorted cell further differentiation could be altered. Since these techniques rely on the availability of a marker, the absence of a surface marker that can separate NSPCs with different differentiation fate renders the purification of subset of cells for therapy impossible.

A live-cell label-free measure of fate potential would solve this problem by reducing the need for specific cell surface markers. Label-free techniques are becoming increasingly more popular for their non-invasive features. Some label free techniques have been developed to identify stem cells from their differentiated progenies based on dielectric properties of stem cells [11] or chemical analysis by Raman spectroscopy [12], [13], [14].

Emerging evidence suggests that energy metabolism and the redox state are important regulators of stem cell functions such as self-renewal, differentiation, lineage-specification and stem cell fate options [15], [16], [17], [18], [19]. Stem cells possess metabolic characteristics that differ from differentiated cells [20], [21], [22], [23]. In the brain, unique features of neurons (e.g. electrical excitability and neurotransmission), oligodendrocytes (e.g. high lipid levels), and astrocytes (e.g. recycling of neurotransmitters and metabolites) also suggest that metabolic requirements of differentiated cells may drastically differ from that of self-renewing, multipotent NSCs. Gene expression analyses have revealed that from development through adulthood, the transition from a NSC/neural progenitor cell to a differentiated neuron, astrocyte, or oligodendrocyte is associated with numerous transcriptional changes, including genes associated with metabolism and energy sensing [24], [25], [26], [27], [28], [29]. Cultured postnatal NSCs also show significantly higher expression of numerous metabolic genes [25], [29]. Noble et al. [19] first observed changes in the intracellular redox state during the self renewal and the differentiation processes of dividing progenitor cells. Only recently a mechanism involved in neuronal differentiation has been identified as requiring SIRT1 activity, which is regulated by nicotinamide adenine dinucleotide (NAD+) and therefore is sensitive to redox state and cell metabolism [30], [31]. Prozorovski et al. [30] showed that redox state does affect the cell-fate decision of NPCs *in vitro*, with oxidizing conditions favoring differentiation into astrocytes, whereas reducing conditions favor neuron formation.

The metabolic coenzyme nicotinamide adenine dinucleotide (NADH) is the principal electron acceptor in glycolysis and electron donor in oxidative phosphorylation. NADH ubiquity renders this coenzyme one of the most useful and informative intrinsic biomarkers for metabolism in live cells and tissues [32]. Since the pioneering work of Britton Chance [33] metabolic imaging of NADH fluorescence levels and of the relative amounts of reduced and oxidized NADH is extensively used to monitor changes in metabolism. NADH has either a short or long fluorescence lifetime component depending whether it is in a free or protein-bound state. Protein-bound NADH is characterized by a complex multi-exponential lifetime decay that has been related to its binding to different enzymes, such as malate dehydrogenase (MDH) and lactate dehydrogenase (LDH) [34]. Metabolic pathways related to carcinogenesis and differentiation are known to change NADH binding sites and enzymatic binding is directly related to NADH cycling through the energy production pathway [35]. For example, Bird et al. 2005 showed that changes in the ratio of free to protein-bound NADH are associated with the NADH/NAD+ redox ratio in breast cancer cells [36]. NADH has been recently used to discriminate different redox ratios of undifferentiated stem cells and their differentiating progenies [37], [38], [39], [40]. We demonstrated that the Phasor approach to fluorescence lifetime microscopy (FLIM) combined with Multi Photon Microscopy (MPM) is a label free and very sensitive method to identify and distinguish different NADH metabolic and differentiation state of germ cells in a living tissue [39], [40].

Here we use the phasor approach to FLIM to measure and identify the metabolic signature of differentiated neurons and NPSCs at different developmental stages. We find that the ratio of free to protein-bound NADH strongly correlates with the differentiation state of the cells and to their fate commitment. Undifferentiated NPSCs have a glycolytic phenotype characterized by high free/bound NADH, while differentiated neurons have an oxidative phosphorylation phenotype, characterized by low free/bound NADH. Here we show that by measuring the metabolic activity and redox ratio of cells we can distinguish neuronal-biased progenitors from glial-biased progenitors. For the first time we demonstrate that NPSCs committed to different differentiation potentials can be identified by their NADH metabolic states even if they are indistinguishable morphologically and by the expression of lineage markers.

## Results

### NADH as Intrinsic Biomarkers in NSC and Neurons

**Figure 1:**
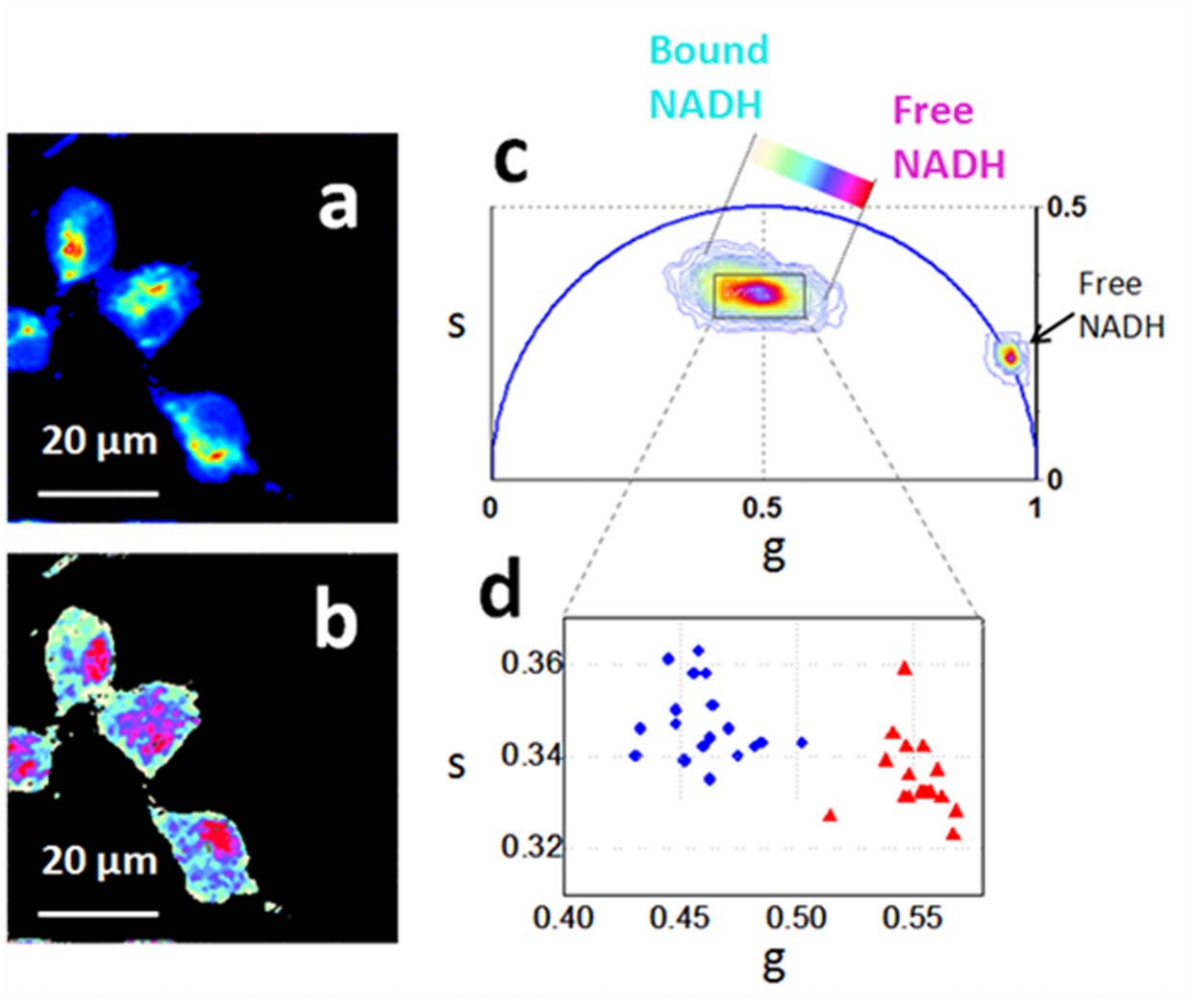
Free and bound NADH distribution within NPSCs. Two-photon fluorescence intensity images (a) and free/bound NADH FLIM maps (b) of the E12 NPSCs excited at 740 nm (c) FLIM phasor plot of E12 NPSCs autofluorescence. A linear cluster represents relative concentrations of free NADH (purple) and bound NADH (cyan-white). Red-purple color indicates a high free/bound NADH ratio, while violet, cyan and white indicate linearly and progressively decreasing ratios of free/bound NADH. (d) Scatter plot of the cell mitochondria (blue circle) and cell nuclei (red triangles). Nuclei contain a higher concentration of free NADH, while mitochondria contain mainly bound NADH.

We perform label free FLIM imaging of the NADH intrinsic fluorescent biomarker within the NPSCs and differentiated neurons. Figure 1 shows a representative image of the NADH autofluorescence from E12 NPSCs. Two-photon fluorescence intensity excited at 740 nm (Figure 1a) highlights NADH distribution within single cells, dim nuclei and bright mitochondria.

We perform the phasor transformation of the FLIM image in which every pixel of the FLIM image is transformed into a pixel in the phasor plot. The coordinates g and s in the phasor plot are calculated from the fluorescence intensity decay of each pixel of the image by using the transformations defined Material and Methods and ref [39] Figure 1c displays the phasor histogram distribution of the FLIM image of E12 NPSCs that is located inside the universal circle of the phasor plot, indicating the multi-exponential characteristic of its decay [39]. The Phasor FLIM signature (Figure 1c) corresponds to the typical complex multi-exponential lifetime distribution of NADH located in the center of the phasor plot. [39] The broad lifetime distribution has a characteristic linear-elongated pattern that reflects a mixture of free and bound NADH, yielding information on different free and protein-bound NADH distribution within a cell. In Figure 1b we map the relative concentration of free and bound NADH within the E12 NPSCs, according to the FLIM phasor location of the free and bound NADH [39]. The nuclei are characterized by a higher concentration of free NADH (purple), while the mitochondria contain predominantly bound NADH (cyan) (Figure 1c and Figure 1d) as previously observed [41], [42].

**Figure 2:**
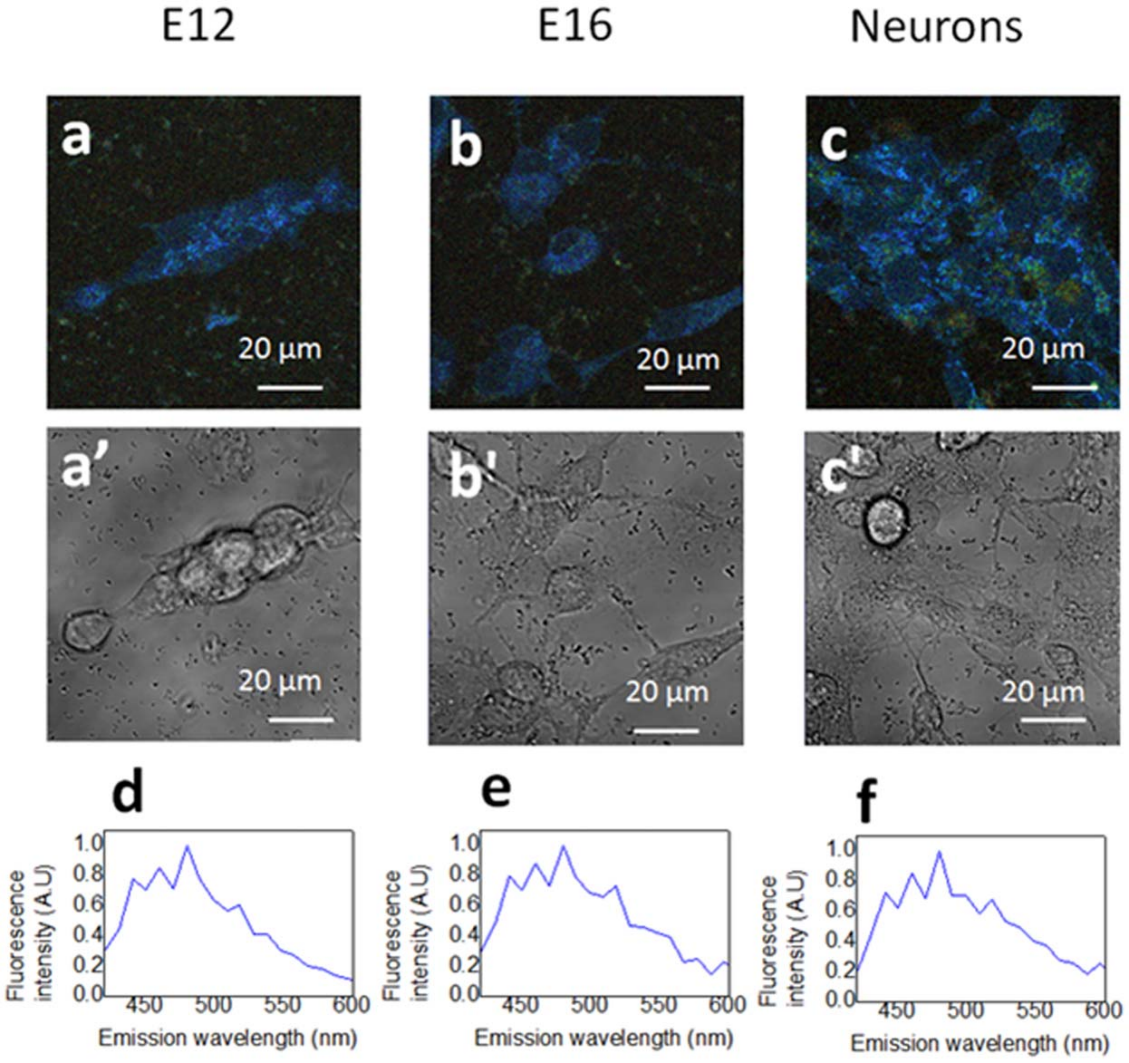
NADH is the major intrinsic source in NPSCs and neurons. (a–c) Spectral images and (a’–c’) transmission images of E12 NPSCs (a), E16 NPSCs (a), and neurons excited at 740 nm. (d–f) Emission spectrum measured in the E12 NPSCs (d), E16 NPSCs (e), and neurons (f) respectively.

The emission spectrum of NPSCs and neurons peaks at 470 nm, confirming that the primary contributor to the autofluorescence signature excited at 740 nm is NADH. (Figure 2).

**Figure 3:**
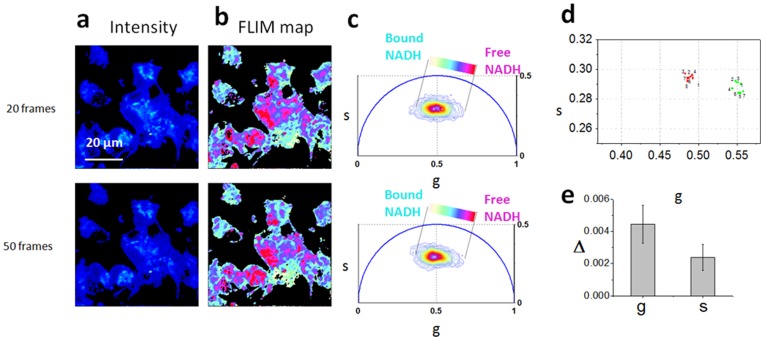
Stability of the Phasor FLIM signature of cells. Two-photon fluorescence intensity images (a) and free/bound NADH FLIM maps (b) of the E12 NPSCs excited at 740 nm for 10–20 frames and 40–50 frames. The same field of view is scanned for a total of 70 frames. (c) FLIM phasor plot of E12 NPSCs autofluorescence. A linear cluster represents relative concentrations of free NADH (purple) and bound NADH (cyan-white). Red-purple color indicates a high free/bound NADH ratio, while violet, cyan and white indicate linearly and progressively decreasing ratios of free/bound NADH. (d) Scatter plot of the average phasor value of two E12 NPSCs that calculated every 10 frames. Numbers indicate the consecutive frames of the scanning. (e) Standard deviations (N_colony_ = 6) of the phasor g coordinates of the cell phasor of single E12 NPSCs.

We verified that the FLIM signature of cells is not affected by photobleaching or photodamage, by scanning 70 frames on the same cells field. (Figure 3).

### Reducing/Oxidizing Conditions and Glycolysis/Oxidative Phosphorylation Shift Cellular Fingerprints Along a Metabolic Trajectory (M-trajectory) between Free and Protein-bound NADH

We performed control experiments on NIH 3T3 fibroblasts and DLD-1 colon cancer cells (See Material and Methods) to show the effect of metabolic drugs on the free/bound NADH relative concentration and to define the trajectory from glycolysis to oxidative phosphorylation in the Phasor plot.

**Figure 4:**
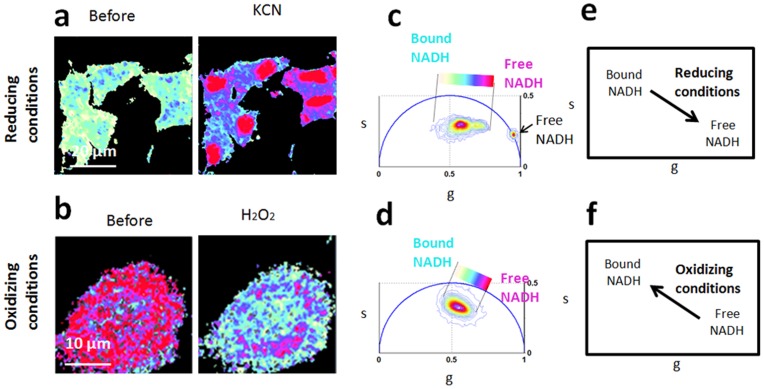
Reducing and oxidizing conditions shift cellular fingerprint along the M-trajectory from free/bound NADH. (a–b) Phasor FLIM color maps at 740 nm of the relative concentrations of free NADH (purple) and bound NADH (cyan-white) NADH of E12 NPSCs before and after the addition of potassium cyanide, which blocks the respiration chain (a) and of NIH3T3 fibroblast before and after the addition of Hydrogen Peroxide (H_2_O_2_), which induced oxidative stress. (b) (c–d) FLIM phasor plot of E12 NPSCs in reducing condition before and after the addition of KCN (c) and of NIH3T3 cells in oxidative stress conditions before and after the addition of H_2_O_2_ (d). Linear cluster represents all possible relative concentrations of free NADH (purple) and bound NADH (white). (e) Schematic diagram indicates that the accumulation of reduced NADH by blocking the respiration chain shifts cellular metabolic signature toward high ratios of free/bound NADH. (f) Schematic diagram indicates that reduction of the fluorescent reduced pool of NADH by oxidative stress induced by hydrogen peroxide shifts cellular metabolic signature toward low ratios of free/bound NADH.

**Figure 5:**
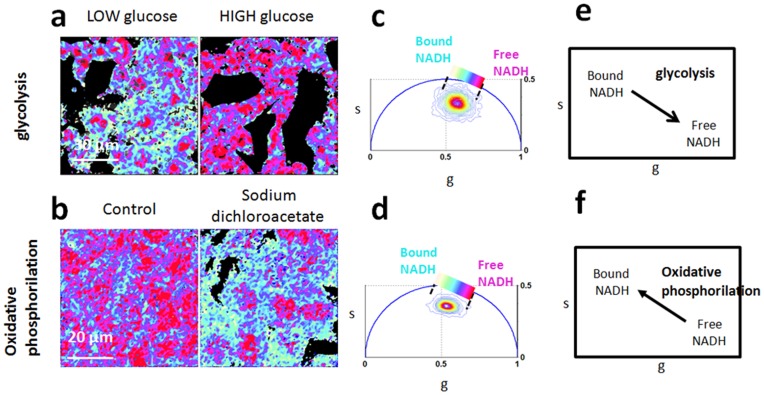
Glycolysis and oxidative phosphorylation shift cellular fingerprint along the Metabolic trajectory from free to bound NADH. (a–b) Phasor FLIM color maps at 740 nm of the relative concentrations of free NADH (purple) and bound NADH (cyan-white) NADH of NIH3T3 fibroblast in low glucose (4.5 mM) and high glucose (22 mM) (a) and of control DLD-1 colon cancer cells and DLD-1 colon cancer cells treated with sodium dichloroacetate (DCA). (b) Dichloroacetate ion inhibits pyruvate dehydrogenase kinase, resulting in the inhibition of glycolysis and a decrease in lactate production. (c–d) FLIM phasor plot of NIH3T3 cells in low and high glucose (c) and control cancer cells and cells treated with DCA.(d) Linear cluster represents all possible relative concentrations of free NADH (purple) and bound NADH (white). Phasor FLIM distribution shifts toward free NADH with an increasing concentration of glucose, while the phasor FLIM distribution shifts toward bound NADH with DCA treatment. (e) Schematic diagram indicates that glucose uptake shifts cellular metabolic signature toward a glycolytic phenotype with high ratios of free/bound NADH. (f) Schematic diagram indicates that the inhibition of glycolysis through DCA treatment shifts cellular metabolic signature toward an oxidative phosphorylation phenotype with low ratios of free/bound NADH.

We identify a metabolic trajectory (the “M-Trajectory”) as the phasor FLIM trajectory from reducing conditions to oxidizing condition (Figure 4) and from a cellular glycolytic phenotype with high free/bound NADH ratios to an oxidative phosphorylation phenotype with low free/bound NADH ratios (Figure 5).

By blocking the cellular respiration with Potassium Cyanide the accumulation of the reduced free NADH within the cell (Figure 4a) shifts the FLIM phasor distribution toward the free NADH phasor location (Figure 4c,e). On the other hand inducing oxidative stress within the cell with 100 µM Hydrogen peroxide (H2O2) the pool of reduced NADH decreases (Figure 5a), hence decreasing the ratio of free/bound NADH and shifting the cellular FLIM signature toward the location of the bound NADH (Figure 5c,e).

Glucose uptake by NIH3T3 fibroblast shifts the metabolic signature toward a glycolytic phenotype with high free/bound NADH ratios (Figure 5a,c,e). On the other hand inhibition of glycolysis in DLD-1 colon cancer epithelial cells shifts the metabolic signature toward an oxidative phosphorylation phenotype with low free/bound NADH ratios (Figure 5b,d,f). Dichloroacetate ion inhibits pyruvate dehydrogenase kinase, resulting in the inhibition of glycolysis and a decrease in lactate production.

Cells treated with DCA are characterized by a lower concentration of free NADH with respect to control cells (Figure 5b). FLIM phasor plot of control DLD-1 colon cancer cells and DLD-1 colon cancer cells treated with DCA.

### NADH FLIM Signature Distinguishes Differentiated Neurons from Undifferentiated NSPCs and Predicts NPSCs Developmental Potential

**Figure 6:**
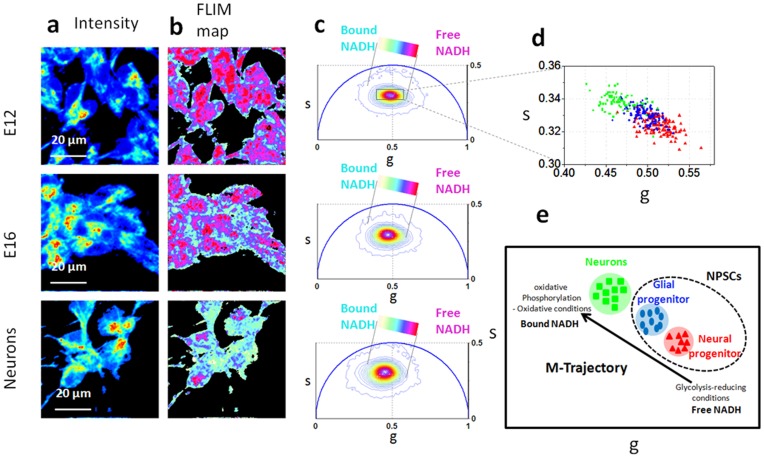
NPSCs and neurons have a unique NADH metabolic fingerprint which predicts developmental stage. Two-photon fluorescence intensity images (a) and free/bound NADH FLIM maps (b) of E12 NPSCs, E16 NPSCs and neurons. (c) Phasor FLIM distribution of E12 NPSCs, E16 NPSCs and neurons. A linear cluster represents the relative concentrations of free NADH (purple) and bound NADH (white). Purple color indicates a high free/bound NADH ratio, while violet, cyan and white indicate linearly and progressively decreasing ratios free/bound NADH ratio. (d) Scatter plot of the cell phasor of E12 NPSCs (red triangles), E16 NPSCs (blue circles) and differentiated neurons (green squares). During differentiation the cell phasor shifts toward the longer lifetime indicating a decrease of free/bound NADH ratio. (e) Schematic diagram of the NPSCs differentiation following a metabolic trajectory (M-trajectory) from free to protein-bound NADH, with a shift from a glycolytic phenotype to an oxidative phosphorylation phenotype.

In Figure 6 we measure the NADH metabolic signature of NPSCs and of differentiated neurons. We show that during differentiation the NPSCs cells follow a precise metabolic trajectory (M-trajectory) associated with free and protein-bound NADH and that metabolic signature of NPSCs predicts their developmental fate toward a neuronal or glial lineage.

We investigate the metabolic signature of NPSCs at different developmental stages. Stem cells predominantly generate neurons at early stages of brain formation and glia at later stages, suggesting the presence of neuron-biased or neuron-restricted progenitors at early stages and progenitor linked to glial fates at later times [43], [44], [45], [46], [47], [48]. From previous studies by Flanagan lab [11], [49] and others, nestin identifies them as NSPCS, while Map2 identifies cells of neuronal morphology as neurons.

NSPCS at different stages of development (E12.5 v. E16.5) are the same size and morphology [50] and currently do not have known markers nor known gene expression patterns to distinguish E12.5 from E16.5. We know they are from this stage of development because we isolated them from embryos of that age (See Material and Methods).

We compared NPSCs from earlier (E12) and later (E16) developmental time points to determine whether differences in the redox state population of neuronal progenitors more prevalent in E12 cells and glial progenitors, which are greater in E16 cells, [31] could be detected by Phasor FLIM of NADH.

**Figure 7:**
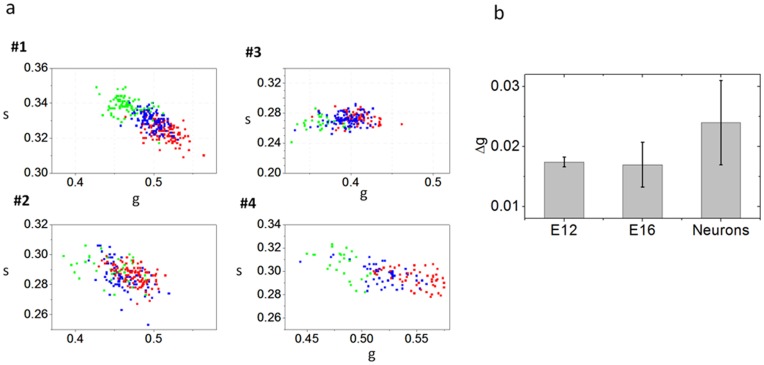
Metabolic heterogeneity of NSPCs and neurons. (**a**) Scatter plot of the cell phasor of E12 NPSCs (red squares), E16 NPSCs (blue squares) and differentiated neurons (green squares) isolated from four different animals. The passage numbers for each experiment are: #1 passage 2, #2 passage 1, #3 passage 7, #4 passage 3. During differentiation the cell phasor shifts toward the longer lifetime indicating a decrease of free/bound NADH ratio. (**b**) Standard deviations Δg (N_animals_ = 4) of the phasor g coordinates of the cell phasor of single E12 NPSCs, E16 NPSCs and neurons.

We use a phasor linear cursor (colored bar in Figure 6c) to represent all possible contributions of the free NADH (purple) and bound NADH (cyan-white). Every single color along the line represents a different relative concentration of the free and protein-bound NADH. The NADH lifetime distribution measured in the NPSCs and differentiated neurons excited at 740 nm is different and the FLIM phasor is distributed along a linear trajectory in the phasor plot that corresponds to the mixture of free and bound NADH (ref [39] ). The FLIM phasor distribution (Figure 6c) and free/bound NADH maps (Figure 6b) show that undifferentiated NPSCs (E12 and E16) are characterized by high ratio of free/bound NADH, while differentiated neurons have a lower ratio. We define “M-Trajectory” (metabolic trajectory) the trajectory from a glycolytic phenotype of undifferentiated NPSCs with high free/bound NADH ratios to an oxidative phosphorylation phenotype of differentiated neurons with low free/bound NADH ratios (Figure 6e). In Figure 6d we measured the phasor FLIM signature of single NPSCs and neurons by calculating the average value of its phasor FLIM distribution by performing manual image segmentation (see material and methods). The average phasor value of cells is calculated within the cursor and plotted in the scatter diagram of Figure 6d. The FLIM phasor values of NPSCs (blue circles and red triangles) are statistically different from differentiated neurons (green squares), (t-test, p<0.0001 Figure 6d and Figure 7) showing a decrease of the free/bound NADH ratio during differentiation.

NPSCs from earlier (E12) developmental time points are characterized by a higher free/bound NADH ratio with respect to later (E16) developmental time points NPSCs (Figure 6a,b,c). Neuronal progenitors in E12 population are characterized by an higher free/bound NADH ratio (Figure 6b,c) indicating glycolytic phenotype and reducing conditions (Figure 6e). On the other hand glial progenitors in E16 population have a lower free/bound NADH ratio with respect to E12, indicating that they are characterized by a more oxidative phosphorylation metabolic phenotype and oxidative conditions. Although the FLIM NADH fingerprint of NPSCs are statistically different at different developmental stages (t-test, p<0.0001 Figure 6d and Figure 7), E12 and E16 show an overlap of their metabolic fingerprints (Figure 6c), indicating the heterogeneity in these populations since E12 cells also contain a minority of glial progenitors while E16 have a small subset of neuronal progenitors.

We observe the same metabolic signature of cells isolated from four different animals. (Figure 7).

### Metabolic Heterogeneity of NSPCs and Neurons

The phasor FLIM analysis at single cell resolution reveals heterogeneity in the metabolic signature and intrinsic free and protein-bound concentrations. We exploit the cell-phasor concept and image segmentation (Ref [39] and material and methods) to measure the phasor FLIM signature of single neurons and NPSCs by calculating the average value of the phasor distribution of single cells. (See Material and Methods).

Figure 6d and Figure 7 show the scatter plot of single E12 NPSCs (red triangles), E16 NPSCs, (blue circles), and neurons (green squares) isolated from four different animals. Metabolic heterogeneity increases during neuronal differentiation, since the cell phasor cluster of undifferentiated NPSCs is smaller in size compared to the cell phasor cluster from differentiated neurons (Figure 7b). The standard deviation of the g component (defined in the Material and Methods) of the cell phasor from an undifferentiated NPSCs is smaller than the one of differentiated neurons (Figure 6c), while there is not a statistically significant difference between the standard deviations of the g components in E12 NPSCs and E16 NPSCs. The fact that E12 NPSCs and E16 NPSCs have similar standard deviation indicates that cell heterogeneity at different developmental stages is not changing significantly. Metabolic heterogeneity of E12 NPSCs and E16 NPSCs reveals their different mixture of neuronal progenitors and glial progenitors with different redox state and hence free/bound NADH. Metabolic heterogeneity of neurons might indicate a different metabolic phenotype of specialized neurons, such as dopaminergic and GABAergic neurons.

## Discussion

In this work we discriminate different differentiation stages of neuronal progenitor stem cells and we identify their differentiation potential by measuring the level of metabolic activity of NPSCs by Phasor analysis and Fluorescence lifetime microscopy of NADH. Our method is label-free and completely non-invasive, thus maintaining cell viability for *in vivo* studies and clinical transplantation.

We identify a metabolic trajectory in the Phasor plot, between a glycolytic phenotype/reducing conditions and oxidative phosphorylation phenotype/oxidative conditions (Figure.4 and Figure 5). Oxidative conditions, such as oxidative stress induced by hydrogen peroxide (Figure 4b,d,f) and the inhibition of glycolysis (Figure 5b,d,f) shift the cellular phasor FLIM signature toward the location of bound NADH. On the other hand reducing conditions, such as the block of the electron chain by Potassium cyanide (Figure 4a,c,e) and glycolysis (Figure 5a,c,e) shift the cellular phasor FLIM signature toward the free NADH.

During neuronal differentiation, the differential change in the concentration of free and bound NADH provides a sensitive readout of the redox state of cells (Figure 6). We map the relative concentration of free/bound NADH in NPSCs at different developmental stages (Figure 6 and Figure7) and we can statistically distinguish the phasor FLIM signature of undifferentiated (NPSCs) from differentiated neurons. (Figure 6d and Figure 7a). Undifferentiated NPSCs have a glycolytic phenotype, characterized by high ratios of free/bound NADH, while differentiated neurons have an oxidative phenotype, characterized by low levels of free/bound NADH (Figure 6). These findings are in agreement with the fact that proliferative cells rely on aerobic glycolysis in contrast to normal differentiated cells which rely primarily on oxidative phosphorylation [51]. During proliferation, the large increase in glycolytic flux rapidly generates cytosolic ATP resulting in high ATP/ADP and NADH/NAD+ ratios [51], [52], [53]. A similar trend in the free and bound NADH ratios has been observed during differentiation of other adult stem cell, such as human mesenchymal stem cells [37], [54] human salivary gland stem cells [55], [56], epithelial cells in the small intestine [57], germ cells [39] and myoblast [58]. To our knowledge this is the first time that a metabolic change in free/bound NADH ratio has been shown in NPSCs and differentiated neurons. We also showed that the FLIM signature of single cells reveal a higher heterogeneity in the metabolic state of differentiated neurons then in undifferentiated NPSCs (Figure 6). Metabolic heterogeneity within neurons might reveal different metabolic phenotype of dopaminergic and GABAergic neurons.

Phasor FLIM not only has the capability to discriminate different metabolic states of undifferentiated progenitor stem cells and differentiated neurons (Figure 6), but also predicts stem cell fate and discriminates between neuronal progenitor cells and glial progenitor cells (Figure 6 and Figure 7). Relatively small changes in intracellular metabolite levels can have profound influences over cell fate decisions and cellular functions [19], [20], [59].

Stem cells with different metabolic rates can be undergoing different fate decisions, but based on morphology and marker expression are indistinguishable from one another**.** NPSCs isolated from different developmental ages (E12 and E16) show no differences in nestin and Sox2 expression. [11] However during embryogenesis, cortical E12 NPSCs are more likely to form neurons and E16 NSPCs preferentially form astrocytes.

The binding of NADH to the transcription factor SIRT1 modulates NSPCs differentiation toward either a neuronal lineage or glial lineage, with oxidizing conditions favor differentiation into astrocytes, whereas reducing conditions favor neuron formation [30], [31]. Therefore free/bound NADH ratios influence cell-fate decision of NSPCs *in vitro*. Remarkably the metabolic signature of E12 and E16 NSPCs are different and the NADH FLIM signature of E12 NSPCs is characterized by a higher free/bound NADH with respect to E16 (Figure 6d). Our data indicate that neuronal progenitors and glial progenitors have a different free/bound NADH ratio (related to the redox state) (Figure 6e) with a higher free/bound NADH ratio for the neuronal progenitor stem cells. E12 which mainly contain neuronal progenitors have a glycolytic metabolic phenotype (Figure 6), characterized by reducing conditions (Figure 4 and Figure 5) in agreement with Prozorovski et al. [30] who showed that reducing conditions favour neuron formation. On the other hand E16 which mainly contain glial progenitors have an oxidative phenotype (Figure 6), characterized by oxidative conditions (Figure 4 and Figure 5) in agreement with Prozorovski et al. [30] who showed that oxidizing conditions favor differentiation into astrocytes. Here we demonstrated for the first time that by measuring the metabolic activity and redox ratio of NPSCs we can predict their commitment to the neural or glial differentiation pathway, independently of the expression of lineage markers.

Phasor FLIM is a promising label-free and non-invasive tool that provides metabolic signatures of NSPCs in* vivo.* This method has the ability not only to distinguish undifferentiated NSPCs from differentiated neurons (Figure 5), but also allows distinguishing, identifying and isolating neuronal-biased progenitors from glial-biased progenitor cells. FLIM enables *in vivo* monitoring of NSPCs metabolic activity, heterogeneity, plasticity and stability, which can be used for isolating cells for transplantation and tissue engineering. Our method could be also be used to monitor in vivo dynamic changes in redox state, oxidative stress and metabolic adaptation that occur in several neurodegenerative diseases such Alzheimer’s and Parkinson’s disease [60], [61]. This technique is very suitable for cell sorting because it is not destructive and does not require exogenous markers or cell treatments that can compromise cell viability. Phasor FLIM represents a powerful method for biophotonics, stem cell biology and regenerative medicine as well as a new platform for cell sorting, high content analysis, metabolomics and drug screening.

## Materials and Methods

### Primary Cell Culture

NPSCs from cortices of CD1 wild-type mouse embryos day 12.5 (E12.5) or E16.5 were cultured as neurospheres as described previously [11].

The passage number of the cells used is between p1 and p7. We used four mice (mothers), one for each experiment. Each experiment was done with cells from a different dissection of multiple embryos. The number of embryos is generally 4–8 per dissection.

Culture media is 1×B27 and N2, 1 mM sodium pyruvate, 1 mM L-glutamine (Invitrogen), 1 mM N-Acetyl-cysteine (Sigma) in DMEM (MediaTech) with 10 ng/ml bFGF (Peprotech), 20 ng/ml EGF (Calbiochem), and 2 µg/ml Heparin (Sigma). For experiments, neurospheres were dissociated with NeuroCult (Stem Cell Technologies) and plated one day prior to analysis on glass bottom dishes (MatTeck) coated with 10 ug/ml poly-D-lysine (MP Biomedicals) and 20 ug/ml laminin (Sigma). The protocol to generate cortical NSPCs from mouse embryos is well established and over 95% of the cells are nestin-positive NSPCs [49].

For neurons, E12.5 NPSCs were plated and differentiated in media without FGF, EGF and heparin for 3–4 days. All NSPCs and neuron cultures were at the same density. NSPCs were always plated 1 day before use at the same density of 100,000 cells per plate (MatTeck-glass bottom 3 mm culture dish). Since the cells divide approximately every 24 hrs they would be at 200,000 per plate the next day. For Neuron generation, 200 K were plated in media lacking growth factors and used after 3 days of differentiation. Since removal of growth factors prevents further division and promotes differentiation they should still be at 200 K after 3 days.

### Cell Treatments

We block the respiratory chain of the DLD-1 colon cancer cells by means of potassium cyanide (KCN) to inhibit the oxidative phosphorylation and increase the mitochondrial concentration of NADH. KCN in PBS was added to the culture medium with a final concentration of 4 mM. Cells were imaged immediately after the addition of KCN. DLD-1 colon cancer cells are treated for 24 hours with 50 mM Sodium dichloroacetate (Sigma #34779) to inhibit glycolysis. NIH3T3 fibroblasts are treated for one hour with Low Glucose (4.4 mM glucose) and High Glucose (22 mM glucose) media. Low Glucose medium (*Invitrogen* Dulbecco’s Modified Eagle Medium (D-MEM), # 11885-092, no Fetal Bovine Serum). High Glucose medium (*Invitrogen* Dulbecco’s Modified Eagle Medium (D-MEM) # 11965-092, no Fetal Bovine Serum). NIH3T3 fibroblasts are treated for one hour with 100 µM Hydrogen peroxide (H2O2) to induce oxidative stress.

### Imaging

Fluorescence lifetime images are acquired a two-photon microscope coupled with a Becker and Hickl 830 card (Becker and Hickl, Berlin). Ti:Sapphire laser (Spectra-Physics Mai Tai) with 80 MHz repetition rate is used to excite the sample. The laser is coupled with a Zeiss Axiovert S100TV microscope. The scanning system is constituted by a scanning mirror (Cambridge Technology Mirror scanner 6350). A Zeiss 40×1.2 NA water immersion objective is used. For image acquisition the following settings are used: image size of 256×256 pixels, scan speed of 32 µm/pixel. A dichroic filter (700DCSPXR, Chroma Technologies) is used to separate the fluorescence signal from the laser light and the fluorescence is detected by a hybrid detector (HPM-100 of Hamamatsu). An additional barrier filter is used to block the near IR light. FLIM data are acquired and processed by the SimFCS software developed at the Laboratory of Fluorescence Dynamics. The excitation wavelengths used were 740 nm. An average power of about 5 mW was used to excite the live tissue. FLIM calibration of the system is performed by measuring the known lifetime of the fluorescein with a single exponential of 4.04 ns. Every FLIM image is acquired over 10 frames of the same field of view.

Spectral images are acquired with a Zeiss 710 microscope coupled to a Ti:Sapphire laser system (Spectra-Physics Mai Tai) and with a 40×1.2 NA water immersion objective (LUMPlanFl Olympus.).

### FLIM Phasor Data Analysis

Every pixel of the FLIM image is transformed in one pixel in the phasor plot as previously described in ref [39], [62]. The coordinates g and s in the phasor plot are calculated from the fluorescence intensity decay of each pixel of the image by using the following transformations:
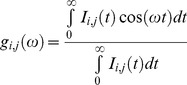
(1)

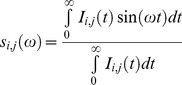
(2)where the indices i and j identify a pixel of the image and ω frequency 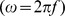., where *f* is the laser repetition rate, i.e. 80 MHz in our experiment. All phasor plots are calculated at 80 MHz, i.e. the first harmonic of the laser repetition rate and for some cases for higher harmonics.

In the phasor plot if the decay is a single exponential 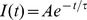 the coordinates are given by:
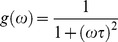
(3)

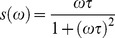
(4)where τ is the lifetime of the decay and ω is the laser frequency. There is a direct relationship between a phasor location and lifetime. Every possible lifetime can be mapped into this universal representation of the decay (phasor plot). All possible single exponential lifetimes lie on the “universal circle” defined as the semicircle going from point (0, 0) to point (1, 0) with radius 1/2. Point (1, 0) corresponds to τ  = 0, while point (0, 0) to 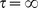. In the phasor coordinates the single lifetime components add directly because the phasor follows the vector algebra. A mixture of two distinct single lifetime components, each of which lie separately on the single lifetime semicircle, does not lie on the semicircle. All the combination of two single exponential components must be along the line joining the two lifetime points. In a system with many single lifetime components the phasor coordinate g and s are described as:



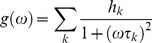
(5)

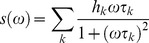
(6)where *h_k_* is the intensity weighted fractional contribution of the single-exponential component with lifetime τ*_k_*. The phasor location of the mixture of single-lifetimes is the intensity-weighted average of the contributions of each single-lifetime that lie separately on the semicircle.

In general in a system with multiple fluorescent components like a tissue the overall decay is a phasor that is the sum of the independent phasors of each fluorescence component:
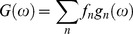
(7)

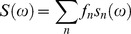
(8)


Where *f_n_* is the fractional contribution of each component characterized by the phasor coordinates *g_n_* and *s_n_*. Two molecular species with multi-exponential decay are identified by two specific points in the phasor plot inside the semicircle. All possible weighting of the two molecular species give phasors distributed along a straight line joining the phasors of the two species. In the case of three molecular species, all the possible combinations are contained in a triangle where the vertices correspond to the phasor of the pure species. The phasor plot of an N-component mixture will be contained in a polygon with N-vertices located in the position of the phasor of each contributing component.

The analysis of the phasor distribution is performed by cluster identification. Clusters of pixel values are detected in specific regions of the phasor plot. The cluster assignment is performed by taking into account not only the similar fluorescence properties in the phasor plot but also exploiting the spatial distribution and localization in cellular substructures or tissues, as described in ref [39]. Fractional intensities of chemical species in every pixel of the image are evaluated with a graphical analysis in the phasor plot as described in ref [62]. We perform image segmentation on the FLIM data by selecting the region of interest of cells within the tissue. The region of interest of cells is selected by using a cursor with arbitrary shape. We calculate the phasor average values within these regions of interest and we represent them in the scatter plot. When measuring the cell phasor, all pixels of the cell (about 1000) are taken in account and the signal to noise ratio of the FLIM signature of cells is higher than in single pixels.

When we calculate the scatter plot of the average phasor value neurons, we select the neurons based on morphology. In ref [11] we showed immunostained images of NSPCs and the neurons that have been differentiated from them (Fig. 2a). In reference [49] we clarified how we identify neurons: To maintain strict guidelines for counting of stained cells, we restricted cells in our analyses to those that met the following criteria: 1) cells counted as neurons expressed the neuronal markers MAP2 or TuJ1, showed no reactivity for the astrocyte and precursor marker GFAP, and had at least one neurite that was longer than the cell body; 2) cells counted as astrocytes were GFAP-positive cells that showed no reactivity for MAP2 or nestin (a marker of NSPCs) and exhibited a filamentous pattern of GFAP reactivity in the cytoplasm.

The t-test is performed on the distributions of different types of the cell average phasor values. All phasor transformation and the data analysis of FLIM data are performed using SimFCS software developed at the LFD.
